# Reducing the occurrence rate of catheter dysfunction in peritoneal dialysis: a single-center experience about CQI

**DOI:** 10.1080/0886022X.2018.1515084

**Published:** 2018-11-06

**Authors:** Jing Hu, Zuoliang Liu, Jun Liu, Hao Zhang

**Affiliations:** a Department of Nephrology, The Third Xiangya Hospital of Central South University, Changsha, China;; b Medical Intensive Care Unit, The Third Xiangya Hospital of Central South University, Changsha, China

**Keywords:** Peritoneal dialysis, continuous quality improvement, catheter dysfunction

## Abstract

To reduce the occurrence rate of peritoneal dialysis (PD) catheter dysfunction caused by catheter displacement or plugging, this study screened all patients with peritoneal dialysis catheterization from 2002 to 2015 from the Third Xiangya Hospital of Central South University. There were 256 patients before continuous quality improvement (CQI) (from 2002 to 2007) and 813 patients after CQI (from 2008 to 2015). The occurrence rate of catheter dysfunction was 5.9% in the preCQI group: seven cases were associated with peritonitis, six cases were involved in omentum wrapping, one case was blocked by oviduct, and one case was blocked by blood clot. Through PDCA (plan-do-check-act) four-step of CQI, the following measures were adopted: (1) Preoperative: treat complications, enema and urine catheterization (2) Intraoperative: strengthen analgesia, Lower the insert position of catheter to 7.5 ∼ 8.5 cm above the pubic symphysis, extending the straight distance of catheter in rectus abdominis and decrease the times of peritoneal dialysis catheter implantation. (3) Postoperative: strengthen the training of nurses, patients and their families. (4) strengthen anticoagulation therapy during peritonitis treatment. (5) use laparoscopic technology for refractory patients, and so on. The occurrence of catheter dysfunction was 1.5% in the postCQI group (*p* < 0.05): two cases were associated with peritonitis, ten cases were involved in omentum wrapping. The measures we adopted in CQI reduce the occurrence rate of catheter displacement or plugging in peritoneal dialysis.

## Introduction

Peritoneal dialysis is a major treatment strategy for patients with end-stage renal disease. Compared with hemodialysis, it has several advantages, such as simple operation, less cost as well as the protection of residual renal function. The key for peritoneal dialysis is to establish a continuous and safe dialysis method. However, in clinic, the catheter-related complications are the common causes for the failure of peritoneal dialysis technique. However, mechanical migration or plugging of catheters are the common catheter-related complications [1].

Continuous Quality Improvement (CQI) is a method of business management concept and system management, which was established and developed by American Deming in 1950s. It was originated from the development of industrialized and standardized management and is a structured and organizational process, which can allow employees to participate in the design of programs and realize the continuous improvement process, in order to provide high quality health services that can meet or exceed people’s expectations. From 1980s to 1990s, the CQI practices were widely applied in all medical institutions in Western countries, and played an important role in the management of the quality of peritonitis [2] and hypertension [3] in patients receiving peritoneal dialysis.

With comprehensive quality management as a foundation and internal and external customer demands as the driving force, CQI can improve the management quality through data collection and quality evaluation while modifying the traditional approaches of post-management retrospective case analysis. Meanwhile, CQI emphasizes that doctors, managers, patients and their families as well as the community should participate in the quality control activities.

In this study, we introduced the concept of CQI into our Department and improved the risk factors and treatment strategies for catheter migration and plugging. Our results showed that the incidence of catheter migration or plugging was significantly reduced after introduction of CQI.

## Patients and methods

### Patients

A total of 1069 cases of inpatients with end-stage renal disease undergoing the catheterization for peritoneal dialysis from 2002 to 2015 were recruited. There were 256 patients (157 males and 99 females with an average age of 49 ± 12 years old) with catheters inserted before CQI (from 2002 to 2007) and 813 patients (505 males and 308 females with an average age of 50 ± 15 years old) with catheters inserted after CQI (from 2008 to 2015). In these 256 patients before CQI, there were 146 patients with chronic glomerulonephritis, 40 patients with high blood pressure benign arteriosclerosis, 38 patients with diabetic nephropathy. For 813 patients after CQI, there were 460 patients with chronic glomerulonephritis, 123 patients with high blood pressure benign arteriosclerosis, 126 patients with diabetic nephropathy. All catheters were the Tenchoff PD straight catheters. The catheters were inserted by the open surgical operation. This study has been approved by the ethnic committee of the Third Xiangya Hospital of Central South University. Informed consents have been obtained from all participants before enrollment in this study.

### Establishment of the CQI team

A CQI team was composed of doctors, nurses in the peritoneal dialysis center together with attending doctors, resident doctors, nursing supervisors as well as the nurses of the dialysis class with their respective responsibilities being clearly defined.

### CQI process

A PDCA four-step (plan, do, check and act) approach was designed during the peritoneal dialysis management process, to improve the preoperative and postoperative processes of the peritoneal dialysis catheterization.

### Design

CQI Team conducted analysis on the cases with the catheter migration and plugging before CQI. Through analysis of the relevant data, the team summarized the possible causes for the catheter migration and plugging. The possible causes were peritonitis combined with omental wrapping, omental wrapping and traction, blood obstruction, plugging of oviduct, simple displacement, etc., which might be associated with the following factors: (1) Poor pre-operative condition of the patients, which affected the operation outcomes; (2) Ineffective local anesthetic drugs, leading to pain which influenced the performance of operation; (3) The position of catheter was too high, resulting in omental wrapping and catheter migration; (4) Repeated intraoperative catheterization, which induced omental wrapping; (5) Ligation to omentum in the purse-string suture; (6) Angle of the dialysis tube was too large and the elasticity caused migration of the intra-abdominal catheter; (7) Non-standard post-operative operation and long drainage time, the rapid drainage rate, leading to excessive negative pressure and subsequent omental wrapping; (8) The patients failed to pay attention to the posture according to the doctor’s advice, leading to the migration of catheter; (9) Not pay attention to the anticoagulant, thrombolytic therapy for the peritonitis anti-infection, causing catheter plugging by fibrin and other inflammatory exudates and eventually omental wrapping.

### Implementation

The implementation of CQI mainly focused on the preoperative preparation, intraoperative catheterization position, surgical approach, and postoperative care, patient education and training. The team members understood their goal and works. The doctors were responsible for the whole process of the draft and timely adjusted the unreasonable processes, and the nurses were responsible for the guidance and intensification of patients. (1) Preoperative: doctors assessed the patients’ conditions, actively corrected preoperative heart failure, infection, hypertension and other complications, defecation and urethral catheterization, and enema treatment when necessary. (2) Intraoperative: Fentanyl was applied to strengthen the effect of analgesia. The dialysis catheterization position was lowered and the position was determined according to the patients’ specific conditions. Especially for patients with large body size and deep pelvic cavity, the catheterization position was appropriately lowered to the intersection of 7.5 ∼ 8.5 cm above the pubic symphysis and 2 cm lateral to the midline; strengthened to improve the intraoperative techniques. The surgeons should be skilled with experiences from at least 100 times of operations. Avoid the repeated operation and the omentum majus irritation in the operation; the purse-string suture should avoid the ligation to the mesentery and omentum majus; Extending the straight distance of catheter in rectus abdominis and reducing the bending angle of the catheter to avoid the migration of intra-abdominal segment of catheter; (3) Postoperative: Strengthened the professional training of nurses for peritoneal dialysis and organized a fixed class for the peritoneal dialysis; strengthened the training of patients and the families, standardized the peritoneal dialysis operations to avoid the long-time or rapid drainage, as well as to avoid the occurrence of enwrapping of omentum caused by siphoning; patients were fasted for 2 d after catheterization operation and the catheter was sealed with 5 ∼ 10 mg of heparin saline every day to make the peritoneal dialysis catheter and the peritoneal environment compatible; encouraged patients to get out of bed early, prevention and treatment of constipation, attention to the rest position, etc.; For the peritonitis treatment, the catheters was sealed with urokinase 1 × 10 4 ∼ 2 × 10 4 U every day until complete remission of symptoms for 7 days. For patients with refractory catheter migration and plugging, conducted the laparoscopic reposition and the fixation of peritoneal dialysis catheters, to solve the problems of recanalization of catheter migration and plugging and found out the reasons, to improve the quality.

### Inspection

The CQI summary was performed once every 3 months. Through follow-up of the patients’ peritoneal dialysis fluid drainage rate, drainage time and properties, the potential adverse factors were corrected and improved accordingly; patients with catheter migration and plugging were treated timely and when necessary, the laparoscopic surgery was performed to identify the reasons followed by data statistical analysis.

### Application

The incidence of catheter migration or plugging was in a declined tendency through CQI, so the CQI was applied in clinic for a long time.

### Statistical processing

The statistical analysis was performed using SPSS 13.0 software. The chi-square test was adopted for comparison of the enumeration data between groups. It was considered statistically significant if *p* < .05.

## Results

Before CQI, there were 256 patients with catheters inserted. After operation, there were 15 patients with poor drainage (Table 1), which was caused by postoperative migration or plugging of catheters, including eight males and seven females, with an average age of 40.9 ± 5.5 years old. There were seven patients combined with peritonitis, including one case with displacement of catheter, which was confirmed by X-ray film, migrating from the true pelvis, with poor treatment effectiveness, and changed to hemodialysis. The other six cases had no displacement of catheters, but one case switched to hemodialysis, and five cases were subject to recanalization after conservative therapy. There were a total of eight patients with non-peritonitis, including five cases with displacement of catheters as confirmed by X-ray film and migrating from the true pelvis. Five patients were corrected by laparoscopic surgery and four of them were found to have varying degrees of enwrapping and plugging of omentum majus in the operation, and one case plugged by fimbriae of uterine tube enwrapping of the end of catheter, and after operation, the catheter was subject to recanalization. Two cases among the non-peritonitis patients were subject to open surgery for catheterization again and the enwrapping and plugging of omentum was found during the operation. One case was combined with abdominal bleeding. Considering plugging by the blood clots, urokinase saline was administrated followed by rapid flushing with heparin saline for thrombolysis. After that, the catheter was subject to recanalization.

**Table 1.** Analysis of patients with catheter displacement or plugging before CQI.

Causes	Cases (*n*)	displacement (*n*)	Conserved therapy (*n*)	Laparoscope (*n*)	Open surgery (*n*)	Result
Peritonitis encapsulans	7	1	7			HD5/PD2
Omental wrapping	6	5		4	2	PD6
Plugging of oviduct	1	1		1		PD1
Blood clot	1		1			PD1

After CQI, there were 813 patients with catheters inserted. After operation, there were 12 patients with poor drainage (Table 2), resulting from postoperative migration or plugging of catheters, including seven males and five females, with an average age of 39.3 ± 6 years old. There were two patients combined with peritonitis, including one case with displacement of catheter as confirmed by X-ray film, two cases undergoing the laparoscopic surgery; one case was withdrawn by the catheter and changed to hemodialysis due to severe adhesion in the abdominal cavity. After management of the infection, the catheter position in one case was adjusted through laparoscopy, which showed smooth drainage after operation. There were ten patients with non-peritonitis, including four cases with displacement of catheters. Ten patients were corrected by laparoscopic surgery and nine of them were discovered to have varying degrees of enwrapping and plugging of omentum majus or mesentery during the operation, one patient was simple displacement and then subject to recanalization after operation. The poor drainage rate caused by migration and plugging of catheters before CQI was 5.9%, which was reduced to 1.5% after CQI, with statistically significant difference (*p* < .05).

**Table 2.** Analysis of patients with catheter displacement or plugging after CQI.

Causes	Cases(*n*)	Displacement(*n*)	Conserved therapy (*n*)	Laparoscope(*n*)	Open surgery(*n*)	Result
Peritonitis encapsulans	2	1		2		HD1/PD1
Omental wrapping	10	4		10	2	PD10

## Discussion

The PD catheter migration and plugging is one of the major catheter-related complications as well as the major reason to cause technical failure and withdrawal of patients. The two-cuff PD straight catheters were adopted for the PD catheterization under surgical operation in our center. Studies have showed that, there is no difference in the incidence of PD catheter migration and plugging between the swan neck catheter and straight catheter [4]. It was reported that the incidence of the PD catheter dysfunction caused by catheter migration or plugging was about 3% ∼ 20% [5, 6]. The rated of inadequate drainage caused by catheter migration or plugging in our center before CQI was 5.9%; although it was not high, it affected the normal peritoneal dialysis, increased the patient's physical and mental suffering; especially in 2007, there were five cases with inadequate drainage, which was caused by catheter migration or plugging, and seriously affected the enthusiasm of the medical staff, therefore, how to reduce the incidence of the catheter dysfunction caused by catheter migration or plugging is the main goal for CQI. Our previous studies have prompt CQI is an effective measure in improving quality of peritoneal dialysis[7, 8], on the basis of the early study we continue to implement CQI to reduce the incidence of peritoneal dialysis dysfunction further and continue to summarize our experience.

Previous studies showed that catheter migration often occurs two weeks after the operation [9]. The catheter migration occurred in our center around 5–16 days after the operation except for patients with peritonitis. The catheter migration is possibly associated with the catheter buoyancy, posture, catheter inserting way, organ affected and the postoperative activity level of patients, etc [10, 11], and one of the major reasons is the very high catheterization position [11]. Since the catheter tip cannot reach the pouch of douglos, which is easy to migrate from the true pelvis. Sun et al. [12] found that, the incidence of catheter migration at a distance 3–6 cm from the PD catheter end to the pouch of douglos was lower than that when at a distance of 6 cm. Currently, according to the Standard Operating Procedures [13], the inserted position of straight catheter is 9–13 cm above the symphysis pubis. However, we found that, the catheterization in the position for patients with large body size and great pelvic anteroposterior diameter may cause unobvious feelings of touching the pelvic floor by guide wire, and patients may have no obviously intraoperative awareness of the defecation, and inadequate drainage may exist during the operation. Li et al. [14] also found that, when the insert position of catheter was located at 8.5 cm above the symphysis pubis, the incidence of catheter migration was decreased significantly. Lowering the insert position of catheter and even cutting the end of TD catheters may lead to reduced incidence of catheter migration and plugging. Considering that the lowering catheter is much easier to be fixed in the pouch of Douglos and the pelvic cavity position is low, the possibility of wrapping of omentum majus is small [15]. Therefore, the insertion position of the catheter should be moved down to 7.5 ∼ 8.5 cm above the symphysis pubis. Therefore, patients will have obviously intraoperative and postoperative awareness of the defecation and no one was withdrawn due to intolerance.

The omental wrapping and traction is also an important reason for poor drainage, which is caused by catheter migration and plugging [16], and can be divided into two cases: combined and not combined with peritonitis. Before 2008, the incidence of catheter dysfunction caused by catheter migration and plugging was 5.9%; and nearly half of patients were combined with peritonitis. Since the intra-abdominal inflammatory substances such as fibrin exudation was increased during the period of peritonitis, which blocks the PD catheter, and inflammation can easily cause omentum and intestinal tube adhesion, and enwrap and plug the catheter, it may cause refractory plugging if timely and effective anticoagulant and fibrinolytic treatment are not administrated. Before CQI, our center mainly sealed the catheter with heparin saline or added to PD dialysate, but it is still prone to plugging by inflammatory substances. After CQI, the catheters are sealed with urokinase with strong fibrinolytic activity, that is, urokinase 1 ∼ 20000 U in 20 mL of normal saline was administrated every day to seal the catheter, and the heparin was added to each bag of PD dialysate until it is clear. After CQI, the results showed that, the proportion of poor drainage caused by peritonitis was dramatically decreased, which only occurred in only two cases.

The patients not combined with peritonitis were complicated. We discovered that, firstly it is related to the catheter insertion position. It was reported that, lowering the position of insertion may reduce the omental wrapping [15]. Secondly, the operation is also very important. The PD fluid drainage time cannot be too long and the drainage rate cannot be too fast, generally no more than 20 min [10]. If the PD fluid drainage is not in a linear form, timely turn off the switch and exclude the factors such as catheter pressure, posture and flatulence impact, etc., since a long time or fast drainage may increase the negative pressure of the end of catheter to attract the omental wrapping of PD catheter. If catheter is not flushed by high pressure and thrombolytic treatment is not performed timely, it is very prone to plugging of the catheters. Before and after CQI, one case was confirmed as wrapping of the end of PD catheters by plugging of fimbriae of uterine tube through laparoscopic examination, suggesting that the negative pressure force of PD catheter is large. Therefore, it is necessary to strengthen the operating training of nurses, emphasize the standardized operation during the postoperative management in CQI link, train the full-time nurses and conduct regular assessment, as well as strengthen the education and training work of patients and families, to avoid the omissions and negligence during the operation. In addition, some patients may be susceptible to omentum irritation. Before CQI, one patient had the plugging of catheter twice. The first time of plugging was combined with migration of catheter, after open surgery, the PD catheter was found to be wrapped by the omentum. In the second time of plugging, it was discovered omental wrapping of catheter with obvious plugging. Considering that it was associated with the omental irritation, the experienced surgeons are required to perform the operation, to reduce the frequency of catheter insertion as well as the omental irritation.

Some studies have shown that the excessive subcutaneous angle of bending of the catheter may cause a large elasticity and subsequently lead to the migration of PD catheter abdominal segment, which is also one of the possible reasons for simple migration. In this study, we only observed one case with simple displacement in our center, probably due to the fact that surgeons had noticed this problem in the catheterization [17].

## Conclusions

In conclusion, through the above actions, the incidence of PD catheter dysfunction caused by catheter migration or plugging was significantly reduced after implementation of CQI in our department from 2008 to 2015, indicating that CQI has effectively reduced the incidence of PD catheter dysfunction. Besides, the catheter migration or plugging caused by omental wrapping is still the important factors for catheter dysfunction, and most of them are induced by peritonitis. In the future work, we should start from the above key points and perform CQI to further reduce the incidence of catheter migration or plugging, as well as to improve the survival and quality of life of PD patients.

## Ethical approval

This study has been approved by the ethnic committee of the Third Xiangya Hospital of Central South University.

Informed consents: Informed consents have been obtained from all participants before enrollment in this study.
